# Subclinical inflammation influences the association between vitamin A- and iron status among schoolchildren in Ghana

**DOI:** 10.1371/journal.pone.0170747

**Published:** 2017-02-02

**Authors:** Abdul-Razak Abizari, Fusta Azupogo, Inge D. Brouwer

**Affiliations:** 1 Department of Community Nutrition, School of Allied Health Sciences, University for Development Studies, Tamale, Ghana; 2 Department of Family and Consumer Science, Faculty of Agriculture, University for Development Studies, Tamale, Ghana; 3 Division of Human Nutrition, Wageningen University, Wageningen, The Netherlands; Institut de recherche pour le developpement, FRANCE

## Abstract

**Background and objective:**

In resource-poor settings, micronutrient deficiencies such as vitamin A deficiency may co-exist with iron-deficiency. In this study we assessed the iron and vitamin A status of schoolchildren and the association between vitamin A and iron status.

**Methods:**

A cross-sectional design using the baseline data of a dietary intervention trial conducted among randomly selected 5–12 years old schoolchildren (n = 224) from 2 rural schools in northern Ghana. Hemoglobin (Hb), serum ferritin (SF) and serum transferrin receptor (sTfR) concentrations were used as measures of iron status. Retinol binding protein (RBP) was used as a measure of vitamin A status. Subclinical inflammation (SCI) was measured using C-reactive protein (CRP) and α_1_-acid glycoprotein (AGP) concentrations. We examined the cross-sectional association between vitamin A and iron status biomarkers with multiple linear regressions.

**Results:**

The proportions of schoolchildren with anemia (WHO criteria), iron-deficiency (ID, SF <15μg/l and/or sTfR >8.5mg/l) and iron-deficiency anemia (IDA, concurrent anemia and ID) were 63.8%, 68.3% and 46.4% respectively. Low or marginal vitamin A status (0.70 μmol/l ≤ RBP < 1.05μmol/l) was present in 48.2% while 37.5% of the schoolchildren had vitamin A deficiency (VAD, RBP <0.70 μmol/l). The prevalence of SCI as well as concurrent VAD and ID were 48.7% and 25% respectively. RBP was associated with Hb (β = 7.2, *P* = 0.05) but not SF (β = 20.7, *P* = 0.33) and sTfR concentration (β = 12.0, *P* = 0.63). In the presence of SCI, RBP was not associated with hemoglobin status but a significant positive association was observed among children without SCI.

**Conclusions:**

The study shows that RBP is significantly associated with Hb concentration but not with SF and sTfR. The observed relationship between RBP and Hb is only significant in the absence of SCI.

## Introduction

Multiple micronutrient deficiencies are common in resource poor settings [1–3]. These micronutrient deficiencies are a result of inadequate consumption of nutrient-rich foods, presence of diseases and inefficient utilization of available micronutrients[4,5]. One of the important vulnerable groups, but often neglected by public health interventions, is school-aged children. Recent studies have emphasized the importance of micronutrient deficiencies among school-aged children as they are particularly vulnerable [3,6]. Iron deficiency (ID) co-exists with vitamin A deficiency (VAD) [6–8]. Concurrent deficiencies of vitamin A and iron have been found among school-aged children in Africa [9,10].

ID is considered one of the ten leading global risk factors with regards to attributable risk [11] and is believed to be an underlying cause of anemia worldwide [11–13]. ID is also known to impair cognitive development of children [14–16]. The long term effect of ID is poor productivity [17,18]. On the other hand, VAD is known to compromise the immune system [19] and is the leading cause of night blindness and a major nutritional determinant of severe infection and mortality among children in the developing world [20,21]. In fact, both ID and VAD increase the risk of morbidity and mortality among young children [22–24]. The work of Marasinghe et al [2] also demonstrated that iron status is also associated with weight-age z-score and vitamin A status is associated with severe stunting. It is hypothesized that VAD causes anemia through 3 mechanisms: modulation of erythropoiesis, reduction of the body’s immunity to infectious diseases thus leading to anemia of infection and modulation of iron metabolism [21,25]. Both observational studies [26–28] and randomized controlled trials [29–31] have reported an association between vitamin A status and iron status. VAD may increase the risk of iron deficient-erythropoiesis and anemia as a result of altering the absorption, storage, release or transport of iron to the marrow [32]. Consequently, interventions that control VAD have been shown to improve iron status and control anemia induced by either ID or infection [33,34]; this has been attributed to the increased absorption and mobilization of hepatic iron stores in the presence of adequate vitamin A [35].

Although ID and VAD are a significant cause of undernutrition, there is a paucity of data on the prevalence of VAD, ID and the association between vitamin A status and iron status among school-aged children in Ghana. Studies on vitamin A and iron status involving different populations are necessary to further elucidate the interaction between vitamin A and iron status. The aim of the present study was to investigate the association between vitamin A status and iron status among rural Ghanaian school-aged children.

## Materials and methods

### Study design

A cross-sectional design using the baseline data of a dietary intervention trial in northern Ghana [36].

### Study area

The study was carried out in Tolon district; one of the 26 districts in the Northern Region of Ghana. The district has a single rainy season beginning in April and ending by October. The vegetation is guinea savannah with a dry season which starts in November and ends in March with maximum temperatures occurring towards the end of the dry season [37]. About 90% of the district is rural and subsistence agriculture is the main occupation of the people. The main staples cultivated include maize, yam and rice. Other crops commonly cultivated and consumed are groundnuts and cowpeas [38]. The main food sources of vitamin A and iron in the district are green leafy vegetables such as amaranth (*Amaranthus spp*.), okra leaves and fruit (*Abelmoschus esculentus*), jute mallow (*Corchorus olitorius*), and kenaf/roselle (*Hibiscus sabdariffa*). Although seasonal, mangoes are also a good dietary source of vitamin A and iron in the district.

### Subjects

The study population consisted of 5–12 years old schoolchildren (n = 241) who were randomly selected from 2 primary schools in 2 rural communities in the Tolon district of Ghana. Data was collected in October 2010 on apparently healthy children; details of the selection procedure has previously been published elsewhere [36]. For this analysis, only schoolchildren with complete data on vitamin A and iron status biomarkers, inflammation biomarkers (C-reactive protein and α_1_-glycoprotein), malaria antigen, anthropometric measurements, dietary diversity and socio-demographic characteristics were included (n = 224).

The dietary intervention trial within which this baseline data was collected was approved by the Medical Research Ethics Committee of Wageningen University, The Netherlands and the Institutional Review Board of Noguchi Memorial Institute for Medical Research, University of Ghana. Permission was also obtained from the district administration, chiefs, opinion leaders of the respective communities and thumb-printed informed consent was obtained from each parent or caregiver

### Biochemical measurements

Serum ferritin (SF), soluble transferrin receptors (sTfR), C-reactive protein (CRP), α1-acid glycoprotein (AGP) and retinol binding protein (RBP) were simultaneously measured using an in-house sandwich ELISA technique [39]. Hemoglobin (Hb) was analyzed with a Pentra 60C^+^ automated analyzer on the same day blood was drawn. Details of the measurements have been previously reported [36]. Anemia was defined using the WHO criteria [12] i.e. Hb <115 g/l for children 5–11 years (n = 213) and 120 g/l for children aged 12 years (n = 11). Using the WHO criteria [12], we further defined ID as SF concentration <15μg/l and/or sTfR concentration >8.5 mg/l (Ramco equivalents) [12,40]. Subclinical inflammation (SCI) was defined as CRP >5 mg/l and/or AGP >1.0 g/l [41]. IDA was defined as concurrent anemia and ID. We also defined VAD as RBP <0.7μmol/l and low or marginal vitamin A status as RBP ≤0.7 but <1.05μmol/l [23]. Concurrent VAD and ID was defined as RBP <0.7μmol/l with ID (SF <15μg/l and/or sTfR>8.5 mg/). The prevalence of ID and VAD were re-calculated after adjustment for inflammation using the correction factors of Thurnham et al [42].

### Malaria screening

The malaria rapid diagnostic cassettes (First Response; Premier Medical) were used to screen for current or recent malaria. The cassette had a sensitivity of 95% and a specificity of 99.5% (First Response; Premier Medical). Children who were positive to the malaria antigen were subsequently treated following recommended guidelines; details of the screening and treatment can be found in our previous work [36].

### Anthropometry

Weight and height of the children were measured according to standard procedures [43]. Height was measured to the nearest 0.1cm with a microtoise (Bodymeter 208; Seca) whilst weight was measured to the nearest 0.1kg with an electronic scale (UNIscale; Seca). The average of duplicate measurements was used to compute z-scores [height-for-age z-score (HAZ), BMI-for-age z-score (BAZ) and weight-for-age (WAZ)] for each child using WHO anthro plus 3.2.2. A verifiable record (birth certificate, health record, community birth register) was used to estimate each child’s age.

### Dietary diversity score

A qualitative 24-hour recall (24hR) was used to assess the dietary intake of the schoolchildren. Mothers and caregivers were first asked to mention all foods including drinks and snacks that were consumed the previous 24 hours (from wake-up to wake-up) preceding the survey by the index child from home and outside of home. She was next probed for likely forgotten foods and then asked to give a detailed description of foods and beverages consumed, including ingredients for mixed dishes. To ensure intake outside home was captured, children were asked to assist their mothers/caregivers in the recall. The 24hR was used to complete the Food and Agriculture Organization’s dietary diversity questionnaire consisting of 13 food groups [44]. In brief, a score of 1 was assigned if a child consumed a food item belonging to a particular food group, else 0. Individual food group scores were aggregated into a dietary diversity score (DDS) for each child. DDS refers to the different number of food groups consumed over a reference period. Any food consumed on multiple occasions from the 24hR was counted only once resulting in a maximum attainable score of 13. The scoring did not consider a minimum intake (in grams) for the food groups.

### Other covariates

Demographic and socio-economic related covariates were assessed with a pre-tested semi-structured questionnaire and included child’s compound size, educational status of household head and mother as well as occupation of household head and mother.

### Statistical analysis

Population characteristics were presented as means (standard deviations) for normally distributed data, median (interquartile range) for skewed data and frequency (percentages) for categorical variables. We analyzed the cross-sectional association between vitamin A status (RBP) and iron status (Hb, SF and sTfR) with hierarchical multiple linear regressions using the General Linear Procedure in SAS. The assumption of normality was assessed with visual inspection (histograms, boxplots and Q-Q plots) and test for normality with the Kolmogorov-Smirnov test. Normality violations were corrected by a natural log transformation of the dependent variables (SF and sTfR) before analysis and the β regression coefficients multiplied by 100 to determine the effect size in percentages. Potential confounders were selected a priori based on literature and included sex [3,8,12], age [3,8,12], SCI [12,41], malaria [12,45], nutritional status of child [8], dietary diversity [3], total family size [33], educational status of household head and mother [33] as well as occupation of household head and mother [8,33]. However, only covariates which had at least a 10% effect on the crude estimate were retained in the complete multivariate models. CRP and AGP were significantly correlated (*r* = 0.61, *P* < .0001; Rho = 0.68, *P* < .0001); although dropping one of the correlated variables is the simplest method, O'Brien [46] recommends using a combined measure of correlated variables as an alternative. We therefore included in our regression models the combined measure of CRP and AGP, SCI (elevated CRP and/or AGP). The Pearson and Spearman correlation coefficients showed no multicollinearity between the other covariates in the regression models. Four multivariable models besides the crude model were formulated. In a hierarchical order, model 1 was adjusted for SCI (dichotomous) and malaria (positive or negative); model 2 was adjusted for demographic factors: age (continuous) and sex (male or female); model 3 was further adjusted for nutritional status (BAZ as a continuous variable) and dietary diversity (DDS as a continuous variable) and model 4 was finally adjusted for socio-economic factors: family size (continuous) and educational status of mother (literate or non-literate). Interaction terms for age, sex and SCI with other covariates (e.g. age*RBP, sex*RBP and SCI*RBP) were included in the models but none was significant. Mathematically, the models were expressed as shown below:

Crude model: y = β0 + β1 × RBP
Model 1: y = β0 + β1 × RBP + β2 × SCI + β3 × malaria
Model 2: y = β0 + β1 × RBP + β2 × SCI + β3 × malaria + β4 × age + β5 × Sex
Model 3: y = β0 + β1 × RBP + β2 × SCI + β3 × malaria + β4 × age + β5 × sex + β6 × BAZ + β7 × DDS
Model 4: y = β0 + β1 × RBP + β2 × SCI + β3 × malaria + β4 × age + β5 × Sex + β6 × BAZ + β7 × DDS + β8 × family size + β9 × Mother’s education

Where y = Hb or log (SF) or log (sTfR)

All statistical analyses were done with SAS 9.3 (SAS Institute Inc., Cary NC.) and a two-tailed *P-*value ≤ 0.05 at 95% confidence interval was considered statistically significant.

## Results

From Table 1, the mean Hb was 109.3 ± 13.4 g/l whilst the median (IQR) for SF and sTfR were 44.8 (29.7 to 93.9) μg/l and 10.1(8.1 to 13.2) mg/l respectively. Overall, 63.8% of the schoolchildren were anemic. Using a cut-off value of <15μg/l for SF concentration, 7.1% of the children had ID and this proportion increased to 8.9% after correction with factors proposed by Thurnham. ID differed widely if based on SF or sTfR (7.1% vs. 68.3%). The overall prevalence of ID defined as SF <15μg/l and/or sTfR > 8.5 g/l was 68.3%.

**Table 1:** Descriptive statistics of the schoolchildren.

Variable	Overall (n = 224)
***Child characteristics***	
Sex (Male); n (%)	141 (63.0)
Age in years; (mean ± SD)	8.1 ± 2.1
DDS (mean ± SD)	5.9 ± 0.9
Family size (mean ± SD)	15.6 ± 8.7
***Iron biomarkers and status***	
Hb concentration, g/l; (mean ± SD)	109.3 ± 13.4
SF concentration, μg/l; median (IQR)	44.8 (29.7, 93.9)
sTfR concentration, mg/l; median (IQR)	10.1 (8.1, 13.2)
Anemia^a^, n (%)	143 (63.8)
ID based on SF <15 μg/l; n (%)	16 (7.1)
ID based on SF <15 μg/l with Thurnham correction [42]; n (%)	20 (8.9)
ID based on SF^b^ <15 μ g/l; n (%)	13 (5.8)
ID based on SF <30 μg/l; n (%)	58 (25.9)
ID based on sTfR >8.5 g/l	153 (68.3)
ID based on SF <15 μ g/l and/or sTfR > 8.5 mg/l; n (%)	153 (68.3)
IDA; n (%)	102 (45.5)
***Vitamin A biomarker and status***	
RBP, μmol/l; (mean ± SD)	0.8 ± 0.2
VAD based on RBP <0.7 μmol/l; n (%)	84 (37.5)
VAD with Thurnham correction [42]; n (%)	69 (30.8)
VAD^c^; n (%)	35 (29.7)
Low or marginal vitamin A (0.7 μmol/l ≤ RBP <1.05μmol/l); n (%)	108 (48.2)
Low or marginal vitamin A after Thurnham Correction [42]; n (%)	112 (50.0)
Concurrent VAD and ID^d^	56 (25.0)
***Malaria antigenemia; n (%)***	182 (81.3)
***Inflammation biomarkers and classification***	
AGP concentration, g/l; median (IQR)	1.0 (0.8, 1.2)
CRP concentration, mg/l; median (IQR)	1.1 (0.3, 4.7)
CRP concentration with malaria, mg/l; median (IQR)	1.3 (0.3, 5.7)
AGP concentration with malaria, g/l; median (IQR)	1.0 (0.8, 1.2)
Elevated CRP (> 5 mg/l)	54 (24.1)
Elevated AGP (>1 g/l)	98 (43.8)
Malaria with elevated CRP; n (%)	46 (20.5)
Malaria with elevated AGP; n (%)	80 (35.7)
Malaria with elevated CRP and/or AGP	89 (39.7)
Presence of sub-clinical inflammation^e^	109 (48.7)
***Nutritional status***	
BAZ; (mean ± SD)	-0.5 ± 0.8
HAZ; (mean ± SD)	-1.4 ± 1.2
WAZ; (mean ± SD)	-1.2 ± 1.0
Stunting; n (%)	69 (30.8)
Underweight^f^; n (%)	26 (16.5)
***Level of education of mother; n (%)***	
Literate	7 (3.1)
***Level of education of father; n (%)***	
Literate	37 (16.5)
***Occupation of mother (n = 221)***	
Farmer	153 (69.2)
Trader	39 (17.7)
Other	29 (13.1)
***Occupation of father (n = 221)***	
Farmer	201 (91.0)
Other	20 (9.0)

DDS, dietary diversity score; Hb, Hemoglobin; SF, Serum ferritin; sTfR, Transferrin receptor concentration; ID, Iron deficiency; IDA, Iron deficiency anemia; CRP, C-reactive protein and AGP, α1 glycoprotein; RBP, retinol binding protein; VAD, vitamin A deficiency; BAZ, Body-mass-index for age z-score; HAZ, Height-for-age z-score; WAZ, weight-for age z-score; IQR, Inter-quarter range

^a^Hb < 115g/l for 5–11 years children (n = 213) and Hb <120g/l for 12 years children (n = 11)

^b^ID based on SF <15 μg/l excluding children with elevated CRP and/or AGP

^c^Vitamin A deficiency excluding children with sub-clinical inflammation

^d^RBP <0.7 μmol/l with ID (SF <15 μg/l and/or sTfR>8.5 mg/l)

^e^Sub-clinical inflammation, elevated CRP and/or AGP (CRP >5 mg/l and/or AGP >1.0 g/l)

^f^underweight was computed for 158 children with age <10 years (n = 66 had ages ≥ 10 years).

We found that 46.4% of the schoolchildren had IDA. The mean RBP concentration was 0.8 ± 0.2 μmol/l with 37.5% of the children being vitamin A deficient; the VAD prevalence decreased to 30.8% after correction with factors proposed by Thurnham. Furthermore, a half (50.5%) of the schoolchildren had low or marginal vitamin A status and a quarter (25%) of them had concurrent VAD and ID. Close to half (48.7%) of the children had SCI. The prevalence of malaria antigenemia was 81.3%; about 40% of those with malaria antigenemia had SCI. The mean DDS of the children was 5.9 ± 0.9. Only a quarter (24.6%) of the children consumed vitamin A-rich dark green leafy vegetables and less than 10% each consumed dairy products, flesh foods, eggs, vitamin A-rich fruits and vitamin C-rich fruits (S1 Fig).

In the crude model of the multivariate linear regression analysis (Table 2), a unit increase in RBP was associated with a significant 10.4 g/l increase in Hb concentration (β = 10.4, *P* = 0.01). After adjustment for possible confounders, the association between RBP and Hb remained statistically significant (β = 7.2, *P* = 0.05). A unit increase in RBP resulted in a non-significant 1% increase in SF in the crude model (β = 0.01, *P* = 0.99). When we adjusted for SCI and malaria, the association between RBP and SF was still not statistically significant (β = 22.5, *P* = 0.29); further adjustment for demography, nutritional status and socio-economic factors did not change the association (β = 20.7, *P* = 0.38). Table 2 also indicates that a unit increase in RBP was associated with a 0.9% decrease in sTfR concentration in the crude model (β = -0.9, *P* = 0.94). This inverse association became positive after adjustment for SCI, malaria and background characteristics but remained not statistically significant (β = 5.7, *P* = 0.63).

**Table 2:** Multivariate linear regression analysis of the association between iron status biomarkers and retinol-binding protein.

Variable	Hemoglobin (Hb)			Serum ferritin (SF)**			Serum transferrin receptor (sTfR)**		
	β	SE (β)	*P*-value	Β	SE (β)	*P*-value	Β	SE (β)	*P*-value
Crude	10.4	3.7	0.01*	0.01	22.8	0.99	-0.9	11.8	0.94
Model 1	8.7	3.7	0.02*	22.5	21.3	0.29	2.9	11.9	0.81
Model 2	7.6	3.6	0.03*	18.3	21.3	0.39	3.8	12.0	0.75
Model 3	7.2	3.6	0.05*	21.0	21.3	0.33	5.3	12.0	0.66
Model 4	7.2	3.6	0.05*	20.7	21.3	0.33	5.7	12.0	0.63

β,regression co-efficient; SE(β), standard error of regression coefficient; n = sample size; Model1: adjusted for sub-clinical inflammation and malaria (elevated CRP and/or AGP); Model 2: further adjusted for age, sex; model 3 was adjusted for Body-mass-index for age z-score (BAZ) and dietary diversity score (DDS) and model 4 finally adjusted for family size and education of mother.

*P-value statistically significant at α = 0.05

**Values were log-transformed and estimates are in percentages.

Tables 3 and 4 show that RBP is only associated with Hb in the absence of SCI. Tables 3 and 4 demonstrate that in the presence of SCI, there is an overestimation of the association between RBP and SF and an underestimation of the association between RBP and sTfR.

**Table 3:** Multivariate linear regression of the association between iron status biomarkers and retinol-binding protein for children with sub-clinical inflammation (n = 109).

Variable	Hemoglobin (Hb)			Serum ferritin (SF)*			Serum transferrin receptor (sTfR)*		
	n = 109			n = 109			n = 109		
	β	SE (β)	*P*-value	Β	SE (β)	*P*-value	Β	SE (β)	*P*-value
Crude	7.7	4.7	0.10	23.3	24.9	0.35	-5.4	14.6	0.71
Model 1	6.8	4.7	0.15	28.1	25.0	0.26	-0.15	14.4	0.99
Model 2	5.6	4.7	0.24	22.8	24.7	0.36	2.3	14.5	0.87
Model 3	4.9	4.7	0.30	25.2	24.5	0.33	4.4	14.5	0.77
Model 4	5.6	4.7	0.24	26.0	25.9	0.32	2.5	14.6	0.86

β,regression co-efficient; SE(β), standard error of regression coefficient; Model1: adjusted for malaria; Model 2: further adjusted for age and sex; model 3 was adjusted for nutritional status (body-mass index for-age z-score/BAZ) and dietary diversity score (DDS) and model 4 finally adjusted for family size and education of mother.

*Values were log-transformed and estimates are in percentages.

**Table 4:** Multivariate linear regression of the association between iron status biomarkers and retinol-binding protein for children without sub-clinical inflammation (n = 115).

Variable	Hemoglobin (Hb)			Serum ferritin (SF)*			Serum transferrin receptor (sTfR)*		
	n = 115			n = 115			n = 115		
	β	SE (β)	*P*-value	Β	SE (β)	*P*-value	Β	SE (β)	*P*-value
Crude	11.7	6.0	0.06	1.3	39.0	0.98	10.7	20.6	0.61
Model 1	12.4	6.1	0.05**	10.3	39.1	0.79	11.1	21.0	0.60
Model 2	12.3	5.9	0.04**	10.8	39.5	0.78	8.5	21.1	0.69
Model 3	12.4	5.9	0.04**	13.0	40.0	0.75	9.3	21.4	0.67
Model 4	11.8	6.0	0.05**	20.0	39.8	0.61	6.4	20.6	0.76

β,regression co-efficient; SE(β), standard error of regression coefficient; Model1: adjusted for malaria; Model 2: further adjusted for age and sex; model 3 was adjusted for nutritional status (body-mass index for-age z-score/BAZ) and dietary diversity score (DDS) and model 4 finally adjusted for family size and education of mother.

**P-value statistically significant at α = 0.05

*Values were log-transformed and estimates are in percentages.

While controlling for all other potential confounders (Table 5), children without SCI had 3.7g/l significantly higher Hb level compared to those with SCI (*P* = 0.04). However, children without SCI compared to those with SCI had a 63% and 0.23% reduction in SF and sTfR concentrations respectively (*P* = 0.001 and *P* = 0.97 respectively). We found a significant positive association between malaria antigenemia (present vs. absent) and SF (β = 25.1, *P* = 0.05) and an insignificant positive association with sTfR (β = 12.8, *P* = 0.09). In contrast, presence of malaria resulted in a non-significant 1.3g/l decrease in Hb (*P* = 0.55). With the exception of sTfR (*R*^2^ = 0.05, *P* = 0.19), the proportion of variance explained by the multivariate models for Hb (*R*^2^ = 0.17, *P* < .0001) and SF (*R*^2^ = 0.20, *P* < .0001) were significant. Lastly, the goodness of fit of the multivariate models as measured by the mean squared errors (MSE) was 12.49, 0.75 and 0.42 respectively for Hb, SF and sTfR.

**Table 5:** Multivariate linear regression analysis for the association between iron status biomarkers and retinol-binding protein.

Variable	Hemoglobin			Serum ferritin			Serum transferrin receptor		
	(Hb)			(log SF)			(log sTfR)		
	Β	SE (β)	*P*-value	β	SE (β)	*P*-value	β	SE (β)	*P*-value
RBP	7.2	3.6	0.05*	20.7	21.3	0.33	5.7	12.0	0.63
SCI (absent vs present)	3.7	1.7	0.04*	-63.0	10.4	< .0001*	-0.23	5.8	0.97
Malaria (positive vs negative)	1.3	2.2	0.55	25.1	13.2	0.05	12.8	7.4	0.09
Age	1.7	0.4	0.0001*	2.1	2.6	0.43	-2.4	1.5	0.09
Sex (male)	0.4	1.8	0.84	15.7	10.7	0.14	4.1	6.0	0.50
BAZ	0.6	1.1	0.59	-11.3	6.3	0.07	-3.7	3.5	0.29
DDS	-2.1	1.0	0.03*	-2.2	6.0	0.72	4.5	3.3	0.18
Education of mother (Non-literate)	-5.5	4.9	0.26	40.0	29.1	0.17	-25.2	16.3	0.12
Family size	-0.2	0.1	0.11	0.4	0.6	0.55	0.2	0.3	0.54

β = co-efficient of regression, SE (β) = standard error of regression coefficient; SCI, sub-clinical inflammation. Note: For Hb, *R*^*2*^ = 0.17, root mean squared error (MSE) = 12.49 and *P* < .0001; for SF, *R*^2^ = 0.20, MSE = 0.75 and *P* < .0001; for sTfR, *R*^2^ = 0.05, MSE = 0.42 and P *=* 0.19

*P-value statistically significant at α = 0.05.

## Discussion

### Prevalence of anemia, ID, IDA and VAD

In this population of schoolchildren from rural northern Ghana, the overall prevalence rates of anemia, ID, IDA and VAD were of severe public health significance [12,47]. In the same area, an earlier work among school children corroborates the severity of anemia, ID and IDA [48]. Overall, the prevalence of anaemia and ID were about twice the rates reported by Herrador et al (anemia = 30.9%, ID = 3.4%) [3] in Ethiopia and higher compared to Righetti et al in Côte d'Ivoire (anemia = 47.3%, ID = 2.7%) [49]. However, the VAD prevalence among this cohort of schoolchildren was similar to that reported by Herrador et al (VAD = 29.3%) [3] in Ethiopia. Using serum retinol concentration, a study among schoolchildren in the Volta region of Ghana reported a similar prevalence of VAD (35.6%) [50] and associated it with inadequate dietary vitamin A intake.

Among this group of schoolchildren, adjusting of RBP with Thurnham et al [42] correction factors or excluding children with SCI had no influence on the public health significance of VAD. Similarly, adjusting SF with Thurnham et al [42] correction had little influence on the prevalence of ID based on SF <15 μg/l. However, we observed that the prevalence estimates of ID varied widely depending on the biomarker used (SF or sTfR); caution may therefore be needed when estimating ID prevalence in a country like Ghana.

In the present study, a quarter of the children had concurrent VAD and ID; a phenomenon described by others[3,6–8,32]. The co-existence of high of ID and VAD could partly be explained by the heavy burden of infections and infestations in the study area, reflected in the high prevalence of malaria and SCI (81.3% and 48.7% respectively). Inadequate intake of ID and VAD-related micronutrients among the school-age children may also partly explain our findings as their dietary pattern was mainly monotonous plant based foods with poor consumption of animal foods as well as vitamin A-rich fruits and vegetables (S1 Fig). Studies have shown that monotonous plant based diets are poor sources of micronutrients due to high concentrations of phytates and other dietary inhibitors in such diets [51,52]. A study among school-aged children in the study area confirms micronutrient inadequacy among the children [48].

### Association between vitamin A and iron status

To our knowledge, the present study was the first to examine the association between vitamin A status and iron status of school-aged children in northern Ghana. The association between RBP and iron status showed a significant result for only Hb (*P* = 0.05) with a unit increase in RBP resulting in a 7.2g/l increase in Hb concentration after adjusting for possible confounders; the present findings corroborate those of other studies [6,28,53]. Several intervention studies have associated vitamin A supplementation with an increase in hemoglobin concentration [30,33,34]. In this regard, suggestions that anemia prevention programmes should include vitamin A improvement programmes are justified. In the present study, we hypothesized a significant positive association between RBP and SF as well as sTfR. However, we found no significant association between RBP and SF nor between RBP and sTfR, a phenomenon described by Hashizume et al [6] and Sales et al [54]. Nevertheless, some studies have reported significant associations between vitamin A status and SF and sTfR [55,56]. Indeed, the association between RBP and SF as well as sTfR has been inconsistent in the literature and may be attributed to extraneous or intrinsic factors within the population that can influence both vitamin A and iron status indicators.

After adjusting for SCI and malaria antigenemia, the β regression coefficients for sTfR and SF increased from to -0.9 to 2.9 and from 0.01 to 22.5 respectively, emphasizing the influence of infection and inflammation on iron status biomarkers. In other words, the findings support evidence that interpretations of the interaction between vitamin A and iron metabolism can be masked by infections which lead to increased SF and sTfR concentrations and decreased plasma retinol concentrations [39,41,42]. Notably, SF has been shown to increase during infection, giving false negative results [41,42,57]; this explains why we observed a significant positive correlation between SF concentration and the inflammation biomarkers (CRP and AGP, data not shown). Thus, the lack of statistical significance between RBP and SF in the present study could be that SCI exerts its impact on SF independent of the vitamin A status of the host.

The prevalence of malaria in the present study was high compared to Otupiri et al (58.6%) in the south of Ghana [58]. Whilst this may suggest a geography variation in the prevalence of malaria even within the same country, studies suggest malaria infection alters the concentrations of iron indicators (notably sTfR) independent of iron status [45,49,59]. Although this may have affected the measured associations in the current study, by mathematically adjusting for malaria, we presume its effect on the iron status indicators was sufficiently accounted for.

### Strengths and limitations of study

In this study, we could not assess the status of other relevant micronutrients such as the B-vitamins, vitamins C and D as well as Zn which have all been linked to iron and vitamin A status [1–3,25]. Hence, though plausible, the co-existence of VAD and ID with other micronutrient deficiencies in our study population which may partly explain our findings cannot be ascertained. In our analysis, however, we controlled for dietary diversity which is known to be a good proxy indicator of micronutrient intake [60].

We used RBP as a proxy measure of vitamin A status rather than serum retinol which may affect our estimates of vitamin A status. Infection, protein malnutrition and inflammation depress RBP concentration because it is an acute phase protein [61,62]. It was thus plausible the RBP levels of the children were not a true reflection of their vitamin A status; but, we presume the multivariate adjustment for SCI and BAZ may have curtailed the influence of protein malnutrition and inflammation on the measured effects. RBP has been shown to correlate well with serum retinol and is a simple, inexpensive tool for assessing vitamin A status in population studies [61,63]In addition, sickle cell traits are prevalent in Ghana [64] and may be associated with increased sTfR [65]; however, because we did not measure hemoglobin variants, we are unable to examine the extent to which these conditions contribute to elevated sTfR in this population. Although residual confounding is often a problem in observational studies even with extensive statistical adjustments [66], we presume this was not a major issue in our present analysis as we had sufficient information on the main potential confounders and adjusted for them. Additionally, high correlations between the predictors may cause large problems in the estimation process [67], but our test for multicollinearity was acceptable in all predictors used.

The major limitation of the present study is its cross-sectional design. Notably, the inference of a possible causality is unfounded since it is not possible to determine whether improved vitamin A status precedes a better iron status. A prospective study would better address this issue.

Finally, the school-aged children we studied may not necessarily be representative of all children in Ghana for two reasons. Firstly, our study population is rural, which limits the generalization of our findings to all school-aged children in Ghana. Secondly, Ghana is a multi-ethnic country with diverse cultural and dietary patterns making it rather impossible to account for all variations among school aged children in Ghana. At best, the present findings can be extrapolated to all rural school-aged children in Northern Ghana where culture and dietary patterns are quite similar.

## Conclusion

The study shows that RBP is significantly associated with Hb concentration but not with SF and sTfR. The observed relationship between RBP and Hb is only significant in the absence of SCI.

## Supporting information

S1 Fig Percentage consumption of different food groups for school-aged children in Tolon district (in October 2010). Vit. A, vitamin A; Vit. C, vitamin C; DGLV, dark green leafy vegetables; YORV, yellow orange and red vegetables; vegs, vegetables.
(PDF)
