# Supplementary material for: Subclinical inflammation influences the association between vitamin A- and iron status among schoolchildren in Ghana
Source: PLoS One. 2017 Feb 2;12(2):e0170747. doi: 10.1371/journal.pone.0170747 (PMC5289472; doi:10.1371/journal.pone.0170747)
Supplement: S1 Fig — Vit. A, vitamin A; Vit. C, vitamin C; DGLV, dark green leafy vegetables; YORV, yellow orange and red vegetables; vegs, vegetables. (PDF) [file pone.0170747.s001.PDF]

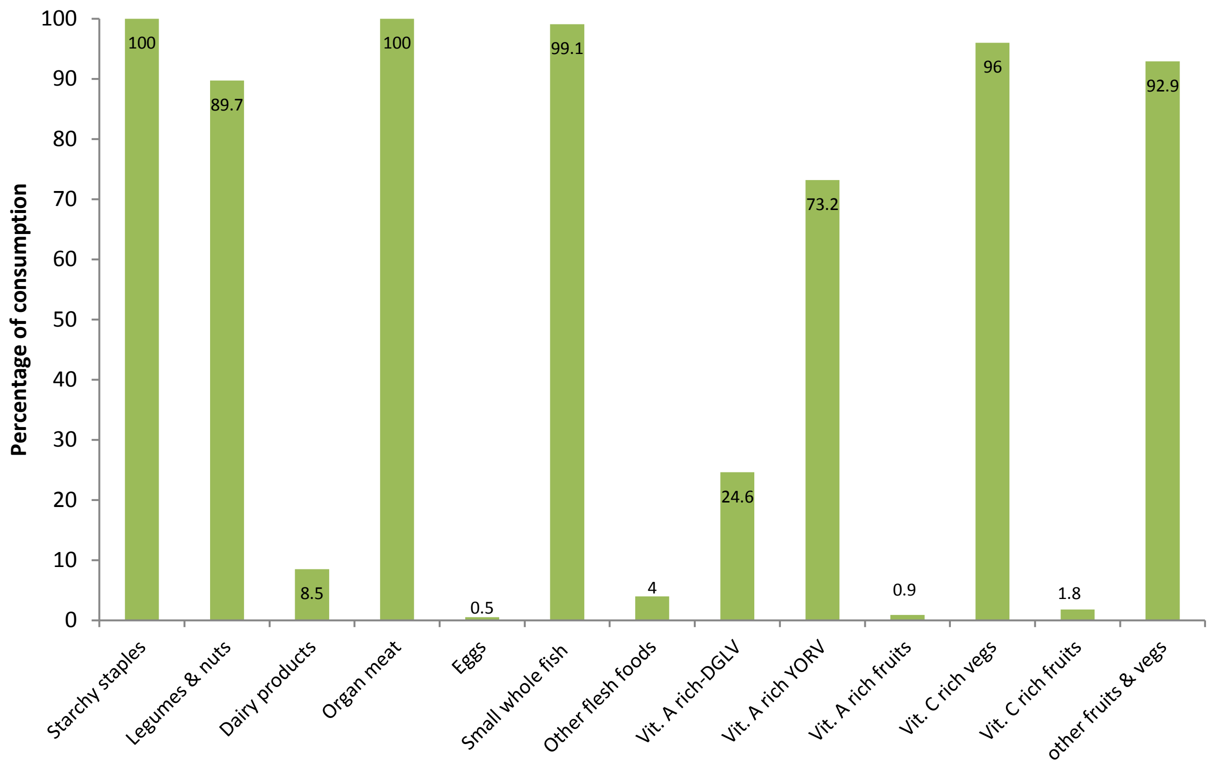

S1 Figure. Percentage consumption of different food groups for school-aged children in Tolon district (in October 2010). Vit. A, vitamin A; Vit. C, vitamin C; DGLV, dark green leafy vegetables; YORV, yellow orange and red vegetables; vegs, vegetables.
